# Noise-Induced Modulation of the Relaxation Kinetics around a Non-Equilibrium Steady State of Non-Linear Chemical Reaction Networks

**DOI:** 10.1371/journal.pone.0016045

**Published:** 2011-01-28

**Authors:** Rajesh Ramaswamy, Ivo F. Sbalzarini, Nélido González-Segredo

**Affiliations:** 1 Institute of Theoretical Computer Science, ETH Zürich, Zürich, Switzerland; 2 Swiss Institute of Bioinformatics, ETH Zürich, Zürich, Switzerland; University of Maribor, Slovenia

## Abstract

Stochastic effects from correlated noise non-trivially modulate the kinetics of non-linear chemical reaction networks. This is especially important in systems where reactions are confined to small volumes and reactants are delivered in bursts. We characterise how the two noise sources confinement and burst modulate the relaxation kinetics of a non-linear reaction network around a non-equilibrium steady state. We find that the lifetimes of species change with burst input and confinement. Confinement increases the lifetimes of all species that are involved in any non-linear reaction as a reactant. Burst monotonically increases or decreases lifetimes. Competition between burst-induced and confinement-induced modulation may hence lead to a non-monotonic modulation. We quantify lifetime as the integral of the time autocorrelation function (ACF) of concentration fluctuations around a non-equilibrium steady state of the reaction network. Furthermore, we look at the first and second derivatives of the ACF, each of which is affected in opposite ways by burst and confinement. This allows discriminating between these two noise sources. We analytically derive the ACF from the linear Fokker–Planck approximation of the chemical master equation in order to establish a baseline for the burst-induced modulation at low confinement. Effects of higher confinement are then studied using a partial-propensity stochastic simulation algorithm. The results presented here may help understand the mechanisms that deviate stochastic kinetics from its deterministic counterpart. In addition, they may be instrumental when using fluorescence-lifetime imaging microscopy (FLIM) or fluorescence-correlation spectroscopy (FCS) to measure confinement and burst in systems with known reaction rates, or, alternatively, to correct for the effects of confinement and burst when experimentally measuring reaction rates.

## Introduction

The workhorse of much research on chemical kinetics has been macroscopic reaction-rate equations. These are deterministic, mean-field descriptions that treat molecular populations as continuous and use macroscopically determined rate constants. Hence they do not always provide an accurate description of reaction kinetics [1], [2]. This lack of accuracy occurs for nonlinear reactions if the population (copy number) of the various chemical species is small enough such that standard errors are not negligible [3]–[9]. These conditions are found, for example, in confined systems that fall short of the thermodynamic limit [10], and in driven reaction systems [11]–[13]. In them, the noise due to molecular discreteness becomes apparent and acquires correlations to give a departure from the behaviour predicted by macroscopic reaction-rate equations [1], [12], [14]–[16].

In this paper we study a representative model of non-linear reaction networks, kept at a non-equilibrium steady state by exchanging input and output with an external reservoir. The input is done in bursts. In a reaction system with burst input 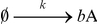 into a reactor of finite volume 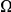 (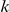 is the macroscopic reaction rate), the variance at a non-equilibrium steady state is 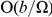 (see Eq. (18) in “Effect of volume and burst on the concentration variance” in “Materials and Methods”). Several environments might host mechanisms of the type burst-input–non-burst-output by non-diffusive, driven processes, such as vesicular traffic in the biological cell [17]. The input–output may be to and from compartments that have physical walls or intersticies caused by excluded volume [18]. In particular, this mechanism occurs in the dynamics of membrane-protein domains (rafts) in contact with a metabolic network [19], [20]. Reaction-rate equations do not discriminate (i) between a stoichiometric (burst) input 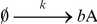 and a non-stoichiometric input 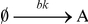, or (ii) the volume 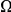 of the compartment.

We account for these effects via chemical master equations, which can be solved using analytical approximations [6], [21], [22] or generating exact trajectories using Gillespie-type stochastic simulation algorithms (SSAs) [23], [24]. We use these tools to study the effects of two noise sources — (i) low copy number as created by finite volume 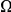 and (ii) input stoichiometry 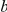 — on the relaxation kinetics of non-linear reaction networks. Specifically, we study the time autocorrelation function (ACF) of concentration fluctuations around a non-equilibrium steady state via its integral (lifetime) and derivatives. For this we use (i) a linear-noise, Fokker–Planck approximation to the master equation via a van Kampen expansion in the system volume [21], [22] and (ii) the full master equation via the partial-propensity direct method (PDM) [24], [25].

We show that the lifetime of chemical species is modulated by burst input *b* and volume 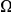 (or confinement 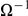). We quantify lifetime by the autocorrelation time of the concentration fluctuations. This autocorrelation is measured in fluorescence-lifetime imaging microscopy (FLIM) or fluorescence-correlation spectroscopy (FCS) [26]. Analysis of FLIM and FCS spectra, however, is based on deterministic reaction rate equations, which are only valid in large volumes and do not reflect the effect of burst input. We show that confinement increases the lifetime of all reactants in a non-linear reaction. Burst either increases or decreases the lifetime. Furthermore, we show that the derivatives of the ACF of the concentration fluctuations are affected in opposite ways by burst 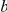 and confinement 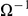, thus discriminating between the two noise source. This directly links the present results to experimental application in two ways: (i) Knowing the lifetime modulation introduced by confinement and burst allows accurately measuring reaction rates in experimental systems. Lifetime is a measure of reaction flux, which is a function of the reaction rates. (ii) Derivatives of the ACF can be used to discriminate between the confinement- and burst-induced effects.

We hence believe that our findings are useful in order to (i) Use FLIM or FCS to measure input stoichiometry 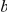 and volume 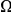 when reaction rates are known. (ii) Correct for the effects of burst input and volume when experimentally measuring reaction rates. (iii) Understand the mechanisms that deviate stochastic kinetics from its deterministic counterpart and choose the right level of description when modelling non-linear reaction networks. (iv) Account for the influences of confinement and burst in formulating coarse-grained governing equations of non-linear reaction models.

We are not aware of previous works tackling the relaxation kinetics of stochastic non-linear reaction networks around a non-equilibrium steady state at arbitrarily low copy number as created by finite volume and driven by a burst input mechanism.

In Section “Model” we introduce the model and its assumptions. Section “Low confinement: the linear-noise approximation” expands the master equation in a van Kampen volume expansion in the linear-noise approximation. From this we study time autocorrelations, which show modulation by the burst 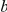 alone. In Section “Beyond the linear-noise approximation: the full master equation”, using the PDM SSA we numerically generate population trajectories of the full master equation as system volume 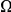 is shrunk and burst 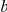 is increased. The autocorrelations of these trajectories have those of the linear-noise approximation as a baseline. Section “Discuss” provides analysis and concludes.

## Results

### Model

As a representative model of non-linear reaction networks out of equilibrium we consider driven colloidal aggregation, for three reasons: First, it is a complete model since this reaction network comprises all three types of elementary reactions: bimolecular, source (input), and unimolecular [27], rendering the results obtained here valid also for other reaction networks. Second, it is a well-characterised model as it has been studied for decades, notably from the 1916 works of Smoluchowski on coagulation and fragmentation. Third, it is a relevant model for many real-world phenomena of practical importance, e.g., in the biological cell (receptor oligomerisation, protein and prion-peptide aggregation, cytoskeletal actin & tubulin polymerisation), in nanotechnology (nano-particle clustering, colloidal crystallisation), in food engineering and the oil industry (emulsion stabilisation, emulsification in porous media), and in metallurgy (dealloying).

We use the chemical master equation to solve the reaction kinetics, neglecting molecular aspects underlying nucleation and growth. Our system is spatially homogeneous (well-stirred) as we disregard structural, spatial, or solvent effects. We also factor out the role of (i) densification upon decrease in system volume, as the total volume fraction is kept constant, and (ii) conformational kinetics, as we do not consider intra-molecular degrees of freedom. In addition, we study our system at a steady state that may be arbitrarily far away from thermodynamic equilibrium as our results do not impose any (semi-)detailed balance condition on the SSA's Markov chain.

Denoting aggregates containing 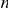 particles as species 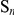 the aggregation reaction network is:
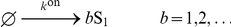


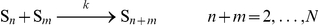


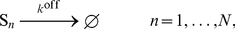
(1)where the 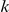
's are macroscopically measurable reaction rates as opposed to specific probability rates [23], [24]. This system describes the aggregation of monomers 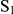 into multimers 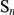 of maximum size 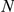. Monomers are input into the finite reaction volume in bursts of arbitrary size 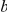. They then form dimers, which can further aggregate with other monomers or multimers to form larger aggregates. Aggregation of multimers happens at a constant rate 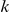 for all possible combinations of multimer sizes 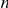 and 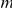. In addition, aggregates of any size are taken out of the reaction volume at constant rate 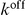, enabling the system to reach a non-equilibrium steady state. For simplicity we consider constant 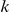
's. The model could readily be generalized to reaction rates 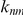 that depend on the aggregate sizes [21], [28]. We chose not to include this generalisation in order to keep the presentation and notation simple, and to establish the baseline effects of volume and burst in the absence of size dependence. Our results will remain valid also in models that explicitly account for size-dependent reaction rates.

If 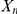 is an extensive variable denoting the number of aggregates of size 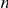 (population of 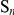) contained in the system volume 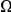, the concentration is 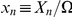. The master equation and its macroscopic counterpart for our model system are then given by Eqs. (20) and (21), respectively (see “Chemical master equation and its macroscopic counterpart for burst-input aggregation” in “Materials and Methods”). We impose that the average total volume fraction 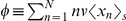 should not vary in time, where 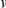 is the volume of each particle and 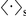 denotes average at steady state. This is satisfied if particle (monomer) influx 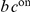 and particle efflux 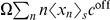 balance each other, where the 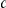
's are specific probability rates, 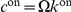 and 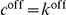. This leads to the mass-balance condition
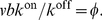
(2)


We isolate the role of 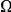 from that of densification by keeping 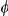 constant as we vary 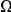 across systems of fixed 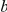, 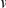, and 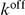. We isolate the role of stoichiometry 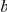 from that of influx 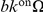 by keeping 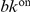 constant as we vary 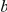 and 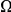 across systems of fixed 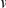 and 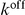. Under mass balance and 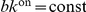., the macroscopic Eq. (21) (see “Chemical master equation and its macroscopic counterpart for burst-input aggregation' in “Materials and Methods”) is insensitive to burst 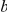 and confinement 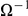 for a fixed 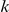. Hence the deviation in our stochastic kinetics from the macroscopic kinetics arises solely due to noise sources 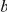 and 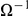.

The master equation associated with the reactions in Eq. (1) provides the time evolution of the probability distribution 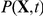 of the population vector 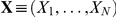 (see Eq. (20) in “Chemical master equation and its macroscopic counterpart for burst-input aggregation' in “Materials and Methods”). We solve it approximately using (i) a van Kampen expansion at the linear-noise, Fokker–Planck level, and (ii) numerically generating exact trajectories of the master equation using an SSA. We compute the ACF of the concentration of species 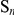 at steady state as
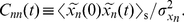
(3)Here, 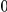 is a time origin at steady state, i.e. after the initial relaxation period 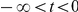, where 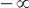 represents an arbitrary origin in the past. 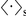 is an average at steady state over time origins and independent stochastic trajectories, 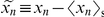 is the fluctuation, and 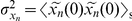 is the variance.

We compute the correlation time of an aggregate of size 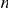 as
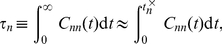
(4)where 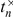 is the first zero crossing. This is a measure of the average decay time and we shall refer to it as lifetime of species 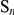. We shall show (in “Low confinement: the linear-noise approximation” in “Results”) that the ACF may become negative due to oscillations, which may make Eq. (4) unsuitable as a measure of a correlation time. The frequency of these oscillations, however, is small enough for our SSA trajectories to justify the approximation in Eq. (4).

We also compute the decay-rate function of the ACF as
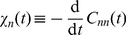
(5)and the initial curvature of the ACF
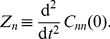
(6)These quantities serve as (curve) characteristics to study the effects of 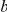 and 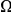 on the kinetics. In addition, they provide a connection with experiments since they can directly be calculated from standard FCS or FLIM read-outs.

In the following, we limit ourselves to a trimer system (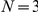) as the simplest aggregation reaction network that comprises all elementary reactions: source reactions, unimolecular reactions, and the two types of bimolecular reactions: homodimerisation and heterodimerisation. This makes the characteristics of the ACF as a function of burst and confinement applicable also for 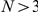 and for other non-linear reaction networks around a non-equilibrium steady state. In our model, we set 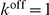, 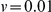, 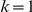, and 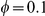. We also limit ourselves to 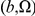-regimes where population fluctuations are not larger than their mean. We estimate the bounds of this regime as follows: The mean number of particles at steady state is 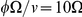. From Eq. (18) we see that the standard deviation at steady state without any aggregation, i.e. for a system containing only monomers, is proportional to 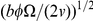 (see “Effect of volume and burst on the concentration variance” in “Materials and Methods”). We impose the mean as an upper bound for twice the standard deviation. This imposes a 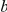-dependent lower bound on the system volume: 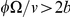.

### Low confinement: the linear-noise approximation

In this section we analytically approximate the master equation associated with the reactions in Eq. (1) by a linear-noise (LN) Fokker–Planck equation [22]. The LN approximation of the master equation is valid at low confinement, i.e., for finite but large enough system volumes. We do this in order to (i) obtain a baseline kinetics on top of which to lay out the full-master-equation kinetics provided in the next section (see “Beyond the linear-noise approximation: the full master equation”), (ii) obtain analytical functions for the ACF, and (iii) reach the large-volume, low-confinement limit where modulation of the ACF by 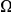 vanishes, thus isolating the dependence on 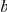.

For the sake of conciseness we provide details of the procedure in “Materials and Methods” (see “Linear-noise approximation of the chemical master equation for burst-input aggregation”). The approximation consists of retaining leading-order terms in a Taylor expansion of 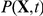 in the small parameter 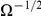. The latter enters after assuming that the noise scales with system volume 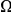 as 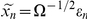, where 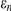 is a random variable evolved by a master equation [15], [21], [22].

In the LN approximation, (i) the noise 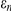 is Gaussian, (ii) the mean 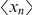 obeys a macroscopic reaction-rate equation, and (iii) the moments of 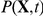, including the ACF, do not depend on 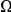 [22]. Despite this, the LN approximation remains useful as there the moments do depend on the burst 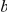, as we show in this section.

For the sake of simplicity we restrict ourselves to 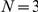. Considering that in the LN approximation the covariances 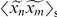 coincide with the second moments 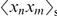 because the mean noise is zero, we solve the time evolution of the first and second moments (See Eqs. (28), (29) in “Linear-noise approximation of the chemical master equation for burst-input aggregation” in “Materials and Methods”) around steady state to obtain the ACF at steady state,
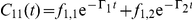


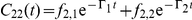


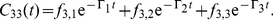
(7)The coefficient 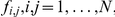 is a ratio of two functions that are linear in the covariances. The rates 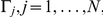 are
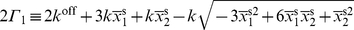


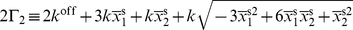


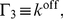
(8)where 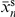 is the steady-state macroscopic concentration of species 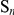 obtained by solving Eq. (21). Note that 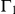 and 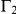 may have an imaginary part, which will give the ACF an oscillatory contribution introducing anticorrelation at late times. By integrating Eq. (7) over 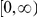 we get the lifetimes,
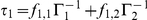


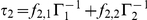


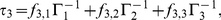
(9)where the integrals of Eq. (7) from their first zero-crossings up to infinity are negligibly small (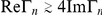). The corresponding integrals over 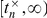 for the SSA-computed ACFs remain small, as mentioned in the Section “Model”.

**Figure 1:**
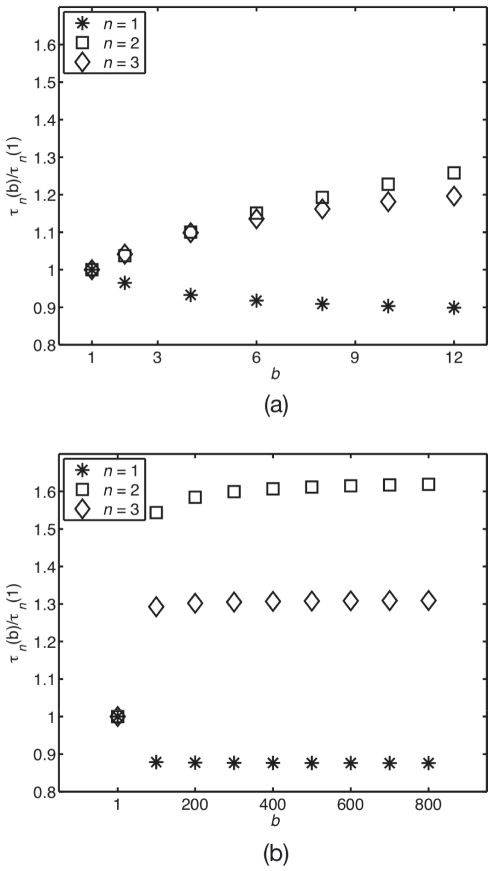
Lifetime from the linear noise Fokker–Planck approximation at low confinement. Lifetime (correlation time) as a function of burst for (a) small and (b) large bursts, normalised to the no-burst, unit-stoichiometry case 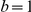 for monomers 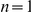, dimers 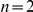 and trimers 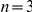. The region above ca. 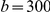 defines the high-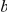 region, where lifetimes become insentitive to 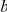. Note that the lifetime of monomer decreases whereas that of the dimer and trimer increases.

The pre-factor 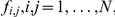 is a ratio of two functions linear in the burst 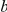 because each covariance is linear in 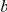. This is seen by solving Eq. (29) (see “Linear-noise approximation of the chemical master equation for burst-input aggregation” in “Materials and Methods”) at steady state under mass balance Eq. (2). As a consequence, 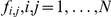, becomes 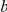-independent at large enough 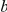, and so do the lifetimes. Figure 1(a) shows how the lifetimes depend on burst. As burst increases from the no-burst case 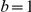, monomer lifetimes decrease and multimer lifetimes increase. As seen from Eq. (9), the lifetimes become 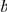-independent at large enough 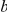, Fig. 1(b). This thus defines a high-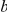 region above 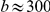. It can also be seen from the general form of Eq. (9) for 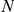 species that, for a non-linear reaction network at a non-equilibrium steady state, 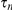 will either increase or decrease with 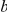, except in zero-measure regions of parameter space where 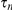 stays constant.

**Figure 2:**
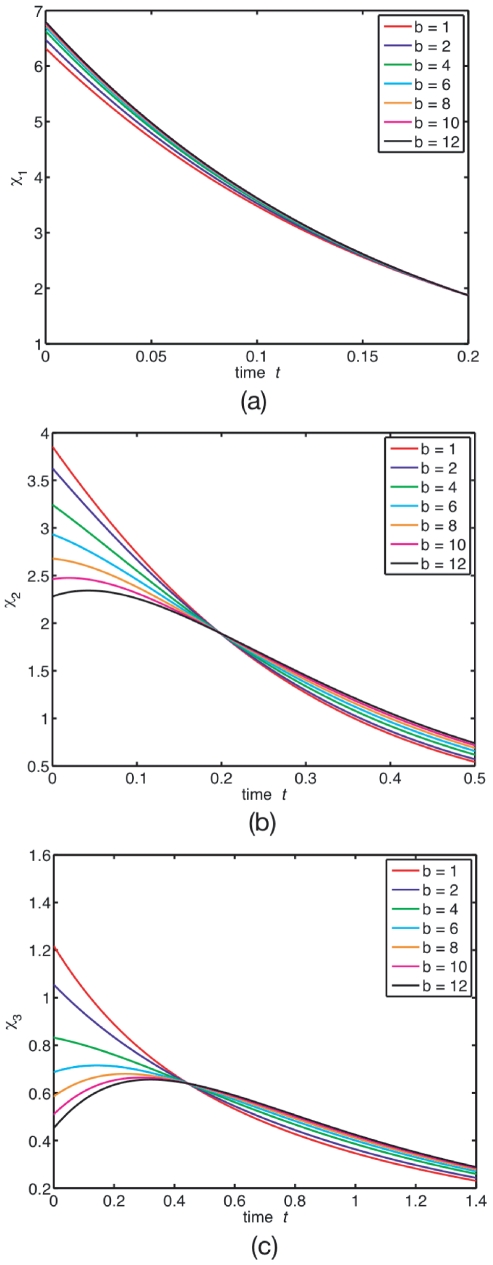
(Colour) Decay-rate function from the linear noise Fokker–Planck approximation at low confinement. Decay-rate function 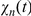 for several burst values 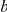. (a) Monomers 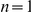. (b) Dimers 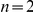. (c) Trimers 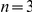. For dimers and trimers there is a threshold burst above which the 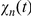 becomes non-monotonic in 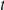. Furthermore, it develops a maximum and it appears at later times with increase in burst 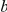.

Figure 2 shows the decay-rate function 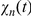 for several burst values. For monomers, 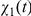 remains monotonic as burst increases, with its maximum at 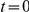. For dimers, 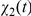 becomes non-monotonic above a threshold burst 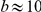, while for trimers the threshold sets in before, at 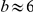. In other words, the decay-rate function of the non-aggregating multimers (trimers) is more sensitive to burst than that of the aggregating multimers (dimers). Note that the maximum that develops shifts from being at 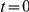 towards later times as burst increases the time 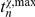 at which 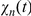 reaches its maximum. We define 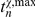 as the time of fastest decay since the (absolute value of the) ACF slope is maximum at this time.

In this section we have calculated the ACF from the linear-noise approximation of the master equation, from which we obtained the lifetimes. We observed that the ACF is a superposition of exponentials with pre-factors modulated by the driving, thereby obtaining the baseline of the burst-induced modulation of the kinetics.

### Beyond the linear-noise approximation: the full master equation

We showed in the previous section how the ACF depends on burst in the low-confinement limit. In this section we show how higher confinement further modulates this ACF. We compute the stochastic trajectories of the populations 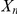 as given by the full master equation to show that shrinking the volume at high-enough confinement further modulates lifetimes and the time of fastest decay. In addition, we introduce the ACF's initial curvature as a further characteristic.

To generate stochastic trajectories from the full master equation we use an efficient SSA [24]. For each parameter set we generate an ensemble of 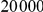 independent trajectories at steady state. Each trajectory is roughly 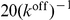 long, about 4 000 time steps of step length 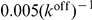. The initial condition for each trajectory is 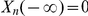, where 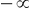 represents an arbitrary origin in the past and 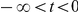 is a period of relaxation to steady state.

### Lifetime

**Figure 3:**
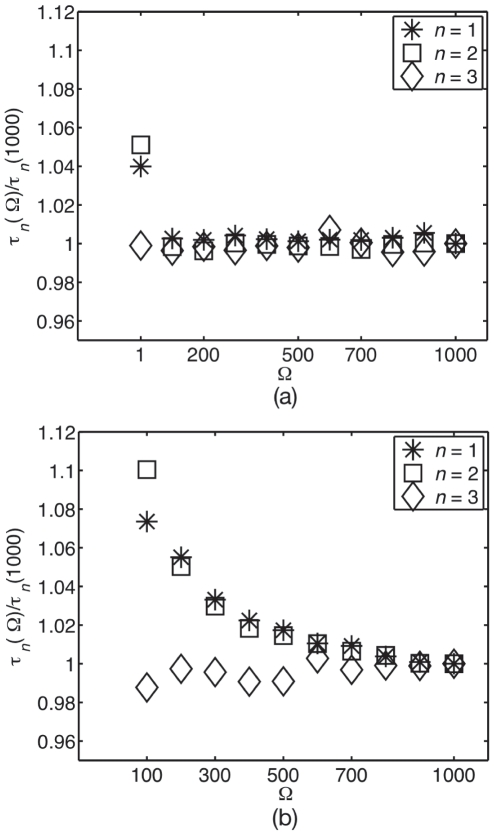
Lifetime from the full-master-equation trajectories. Lifetimes as a function of system volume 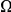 for constant burst 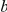, each normalised to its corresponding 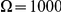 system. (a) No burst, 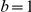. (b) Higher burst, 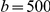 for monomers 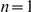, dimers 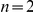 and trimers 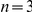. Note that the system becomes insensitive to 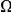 at large enough 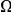, as the linear-noise approximation predicts (see “Low confinement: the linear-noise approximation” in “Results”). As volume decreases, the system departs from linear-noise behaviour. Note that trimers are insensitive to volume as they are not a reactant in a non-linear reaction.

Figure 3 shows the lifetimes 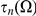 as a function of volume 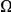 for both no burst 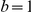 and a burst value in the high-burst region observed in the LN limit, 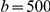. We see that shrinking 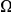 increases 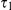 and 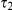, but not 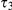, and that this effect is more appreciable at larger 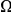 as burst 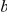 increases.

**Figure 4:**
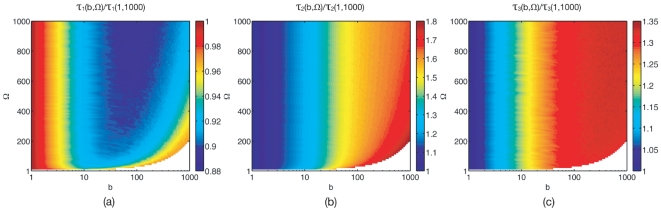
(Colour) Lifetime from the full-master-equation trajectories. Lifetimes normalised to their value at 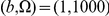. (a) Monomers 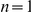, (b) dimers 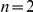, (c) trimers 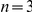. N.B.: The void region for small 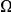 corresponds to population fluctuations becoming larger than the mean. Shown is an interpolation of data sampled at intervals 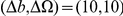.

Figure 4 shows maps of lifetime versus volume for a burst range. The trimers' map shows that volume does not affect lifetime, as also seen in Fig. 3. Figure 4 shows that for monomers and dimers, increasing burst 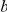 extends the 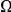-interval over which the lifetime varies with 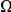. This can also be seen in Fig. 3. In other words, burst seems to act as an amplifier (multiplicative-noise parameter) for confinement-induced lifetime modulation.

The monomer lifetime 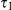 deserves special attention because it is the only lifetime that is non-monotonic in the burst 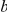, see Fig. 4(a). For any 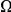 fixed in the interval 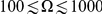, 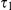 decreases with 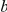 and then increases back for 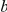 beyond some threshold 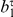. The threshold 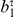, in turn, decreases with confinement 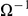. The non-monotonicity of 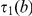 is a high-confinement effect because it does not occur in the linear-noise Fokker–Planck limit, see Fig. 1. The existence of the threshold 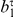, nonetheless, is not surprising because for monomers, confinement and burst cause opposing modulations: confinement *increases* lifetime whereas, as seen from the LN limit, burst *decreases* it. Since burst amplifies the confinement-induced modulation of the lifetime, it acts as a 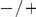 switch for it.

We can also view the problem from the perspective of how confinement affects burst-induced lifetime modulation: varying 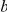 while we fix 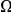 below the LN limit, see Fig. (4). In other words, by looking into a hypothetical volume-dependent, high-confinement version of Eq. (9). Note also that the lifetimes 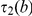 and 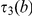 are the only lifetimes increasing with burst 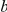 in the LN limit. Recall that further confinement 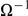 allows the decreasing function 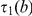 to acquire a slope of the same sign of that of 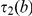 and 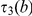 for large enough burst 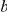. This suggests that confinement 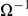 is an amplifier of burst-induced lifetime modulation. This amplification, in turn, must result from 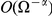 terms entering 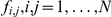, and/or 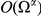 terms entering 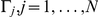, in Eq. (9) for some 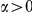.

In summary, we have shown that confinement 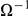 increases the lifetime of all species that are reactants in a bimolecular reaction, i.e., trimers are insensitive to confinement. Confinement-induced modulation lays on top of the burst-induced modulation seen in the LN limit. It provides an effective modulation that may lead to non-monotonic behaviour.

### Derivatives of the ACF

**Figure 5:**
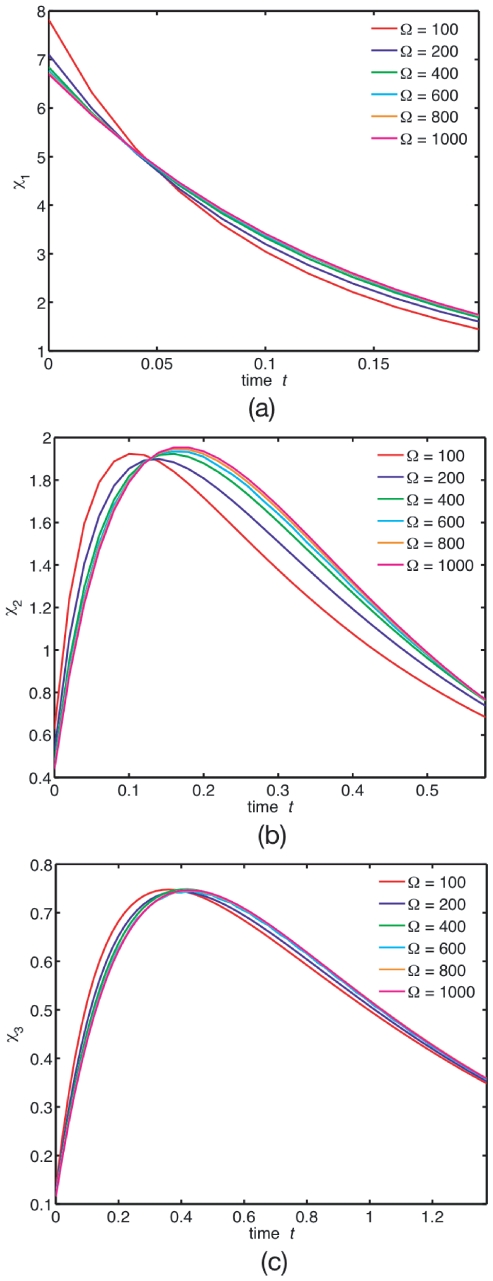
(Colour) Decay-rate function from the full-master-equation trajectories. Decay-rate function 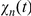 for (a) monomers 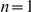, (b) dimers 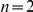, and (c) trimers 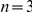 as volume shrinks at 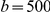. 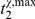 is defined as the position of the maximum. Shrinking volume alone reduces 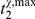, as opposed to increasing 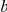, see Fig. 1. Similar trend is also shown by the trimers.

Figure 5 shows representative samples of how the decay-rate function 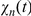 responds to volume shrinking at burst 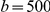. This burst value corresponds to a monotonicity post-threshold value for the multimers (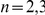) at low confinement, see Fig. 2. Our aim here is to study how confinement alters this low-confinement behaviour. We look for qualitative features that correlate with changes in volume 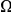 and stoichiometry 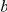. These features may possibly be used to develop quantitative methods to characterise local volume and stoichiometry from FCS-sampled ACFs.

From Fig. 5 we can see that for monomers, 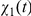 is monotonic. For multimers (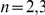), 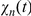 is non-monotonic, making 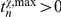. This change in monotonicity is a purely burst-induced modulation, as opposed to confinement-induced, and exists already in the LN limit (see “Low confinement: the linear noise approximation”). Note that confinement reduces 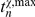, as opposed to burst, which increases it, see Fig. 2.

Up to now we have studied two-dimensional datasets 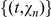. To facilitate feature detection in an FCS experiment, it would be desirable to reduce dimensionality from two dimensions to one. To this end we now study the ACF initial curvature 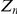. Since 
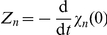
, from Fig. 5 we see that 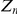 is monotonic for all species as the volume shrinks.

**Figure 6:**
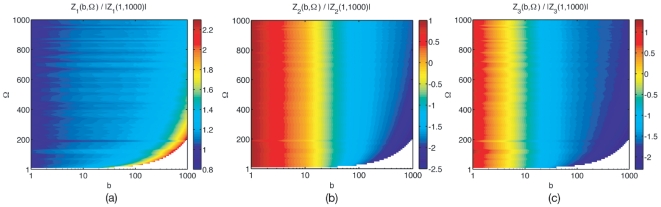
(Colour) ACF initial curvature from the full-master-equation trajectories. ACF initial curvature, 
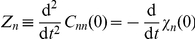
, normalised by its absolute value at 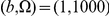. (a) Monomers 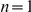, (b) dimers 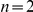, (c) trimers 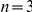. This quantity serves as a lower dimensional read-out of the decay-rate function 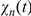. N.B.: The void region for small 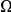 corresponds to population fluctuations becoming larger than the mean. Shown is an interpolation of data sampled at intervals 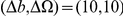.

Figure 6 shows the ACF initial curvature 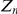 for burst and volume ranges. For monomers, confinement *increases* 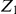, more noticeably at larger burst. Moreover, 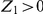, reflecting the monotonicity of 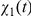. For multimers (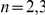), on the contrary, confinement *reduces* the ACF initial curvature from a positive to a negative value as we go from the small-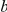
–large-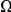 region to the large-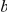
–small-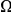 region. This reflects the non-monotonicity of 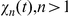, beyond a burst threshold. In other words, the change of monotonicity is a purely burst-induced modulation also at high confinement. There is no qualitative difference between aggregating (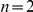) and non-aggregating (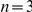) multimers.

## Discussion

In Table 1 we summarise the behaviour of the most relevant characteristics we studied, which can be obtained *a posteriori* from standard FCS or FLIM read-outs. This table may serve as a reference for contrasting burst-induced and confinement-induced modulations and be useful for later studies of the mechanisms behind them. An immediate use may be to help discern whether the noise source is burst-induced or confinement-induced.

**Table 1:** ACF characteristics upon increasing burst 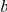 and confinement 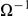.

Characteristic	LN approx.	Full master equation
	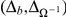	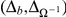
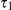	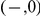	(⌣, +)♦
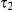	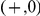	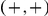
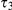	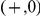	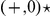
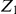	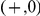	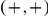
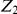	(−,0)▴	(−,+)▴♠
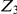	(−,0)▴	(−,+)▴♠
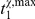	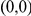	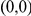
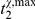	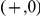	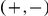
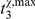	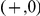	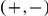

Characteristics upon increasing burst 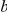 and confinement 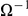, encoded as pairs 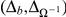, where 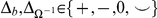 is the modulation of the relevant characteristic as 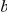 or 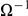 increases, respectively, while keeping the other constant. Here 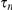 is the lifetime, 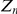 is the initial curvature of the ACF and 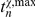 is the time at which the decay rate of the ACF is maximum for monomers 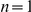, dimers 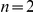 and trimers 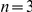 (see “Model” in Section “Results”) The modulation states are positive (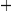), negative (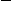), negligible or zero (0), and decreasing-then-increasing (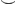). ♦: 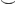 because there exists a competition of burst-induced versus confinement-induced modulation. 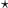: 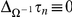 for species reacting only unimolecularly. ▴: 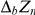 decreases from positive to negative, reflecting the role of burst in changing 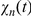 monotonicity. ♠: 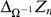 does not change sign, hence 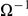 does not change 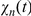 monotonicity.

The presence of oscillations implies that care must be taken when calculating lifetimes. We have calculated them by integrating the ACF up to its first zero crossing. This is only justified if the frequency of the oscillations is low enough, as is our case, see Eq. (4). For reaction networks showing non-negligible frequencies, calculating lifetimes as the mean of the lifetime distribution could be considered. This distribution could be obtained from the distribution of the so-called “time to the next reaction”, as generated by the SSA [23], [24], however requiring a suitable definition for lifetime as a function of it.

Finally, including scission as a backward reaction in Eq. (1) would not modify the qualitative behaviour presented in this paper. This is because scission is a unimolecular reaction, whose reaction degeneracy, and hence its propensity, is linear in the population while the degeneracy for aggregation is non-linear [23], [24]. Consequently, scission would modify the populations at the same rate for all reactants 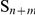 and would not introduce any additional non-linearities. This is also confirmed by SSA simulations (data not shown). Note that scission is not negligible for aggregates of low enough interfacial tension, whose equilibrium in the absence of driving is not totally displaced to the right.

In summary, we have characterised fundamental properties of the relaxation kinetics of a non-linear stochastic reaction network around a non-equilibrium steady state. We have chosen as a model a confined, open colloidal aggregation system of finite volume 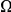. The system is driven by a monomer influx in bursts of 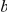 monomers and a non-burst multimer outflux. Specifically, we studied the trimer aggregation network as the simplest aggregation network comprising all types of elementary reactions. This makes our observations on the relaxation kinetics applicable also to larger aggregation networks and to other non-linear reaction networks around a non-equilibrium steady state. We studied the role of (i) low copy number created by confinement 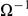 at constant volume fraction, and (ii) burst influx 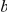. Both of these are noise sources that increase concentration fluctuations.

We accounted for these stochastic effects using (i) a linear-noise, Fokker–Planck approximation, valid in the low-confinement limit, and (ii) exact trajectories of the master equation from a stochastic simulation algorithm, modelling high confinement. We used the time autocorrelation function (ACF) of species concentrations to study the relaxation kinetics towards the non-equilibrium steady state.

We have proposed the following curve characteristics to study the response of the ACF of a species 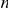 to confinement (inverse volume) and burst: (i) the lifetime 
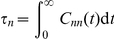
, (ii) the decay-rate function 
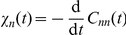
, and (iii) the ACF's initial curvature 
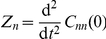
.

We observed that increasing burst 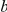 monotonically increases or decreases the lifetimes of all species, except in zero-measure regions of parameter space where they stay constant. On the other hand, confinement 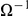 increases the lifetime of those species undergoing bimolecular reactions (monomers and dimers), but does not modulate those undergoing only unimolecular reactions (trimers). This can lead to a competition between confinement-induced and burst-induced modulations. From these observations we hypothesise that the ACF is modulated through terms of the form 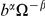 for some 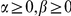.

Burst alone is responsible for making 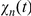 non-monotonic for some species. The peak in the non-monotonic 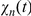, reflected by 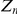, is shifted in opposite directions by burst 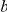 and confinement 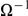.

We believe that our results are useful to measure volume and burst in systems with known reaction rates, or, alternatively, correct for the effects of volume and burst when experimentally measuring reaction rates using fluorescence-lifetime imaging microscopy (FLIM) or fluorescence-correlation spectroscopy (FCS). Furthermore, our results help understand the mechanisms that deviate the stochastic kinetics of non-linear reaction networks at high confinement and burst from their deterministic counterpart.

## Materials and Methods

### Effect of volume and burst on the concentration variance

Consider the following chemical reaction
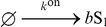


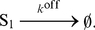
(10)Also consider the step operator 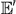 acting on a function 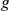 of the population 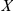 of 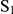 such that 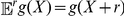. The master equation for the stochastic evolution of reaction (Eq.10) can then be written as
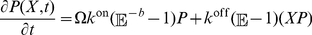
(11)where 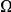 is the volume in which the reaction takes place and 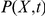 is the probability distribution for having 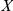 molecules of 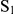 at time 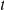.

Multiplying Eq. (11) by 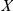 and summing over all possible values of 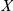 we get the evolution of the mean
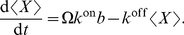
(12)We obtain the steady-state mean by setting the time derivative to zero
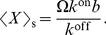
(13)By multiplying Eq. (11) by 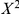 and summing up over all possible values of 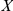 we get
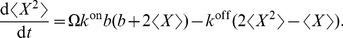
(14)By setting the time derivative to zero we see that at steady state
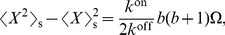
(15)which is the population variance. Hence the variance of the concentration, 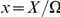, at steady state is
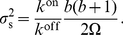
(16)Note that 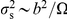.

Imposing that the average volume fraction 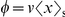 is constant at steady state, where 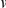 is the volume of a monomer, leads to the mass-balance condition
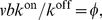
(17)see Eq. (2). Fixing 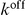, 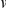, and 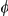 hence fixes the product 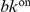, which appears in the macroscopic rate equation. The condition (Eq.17) leads to the concentration variance
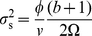
(18)and the mean concentration
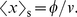
(19)Imposing mass balance thus modifies the scaling of the steady-state variance to 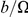.

Having a non-linear reaction in Eq. (10) would leave this scaling unchanged as long as the mass-balance condition holds.

### Chemical master equation and its macroscopic counterpart for burst-input aggregation

The master equation associated to reactions (Eq. 1) is:
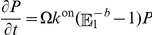


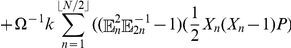


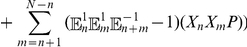


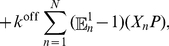
(20)where 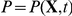 is the joint probability distribution of the population vector 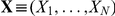 at time 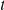. 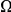 is the volume of the reaction compartment and 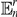 is the step operator acting only on functions of 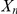 such that 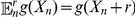, where 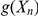 is some function of 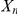. Note that imposing that the average volume fraction 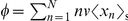 is constant at steady state, where 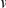 is the volume of a monomer, again leads to the mass-balance condition in Eq. (17).

The macroscopic (i.e., deterministic) counterpart of Eq. (20), valid in the limit of very large volumes [10], is given by
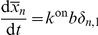


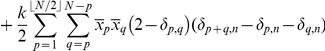


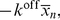
(21)where 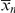 is the macroscopic concentration of the aggregate of size 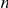.

### Linear-noise approximation of the chemical master equation for burst-input aggregation

Analytically solving Eq. (20) for 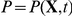 is intractable since the right-hand side of the equation is non-linear in the populations. This is almost always true for systems involving bimolecular reactions, save very few exceptions. We can, however, follow van Kampen to volume-expand the master equation in the small parameter 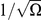 [15], [21], [22].

We consider the stochastic quantity 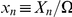 to fluctuate around the mean *macroscopic* concentration 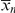. This is satisfied by the following ansatz
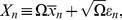
(22)where 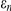 is random, and of non-zero mean in general. From Eq. (22) we see that any function of 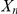 satisfies 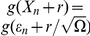, which allows introducing the volume expansion
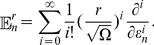
(23)From Eqs. (22) and (23), Eq. (20) becomes
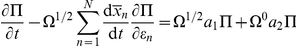


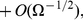
(24)where 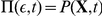, 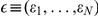, and 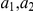 are differential operators.

To make Eq. (24) a proper expansion in 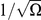 we impose that terms proportional to 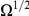 on both sides are equal. Subsequently, equating terms proportional to 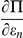 gives Eq. (21). Then, at 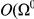), we are left with
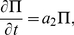
(25)where 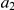 is the operator
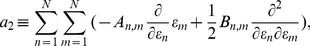
(26)with 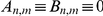 if 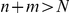, otherwise
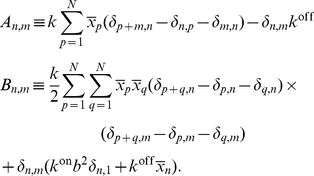
(27)Equation (25) is a linear Fokker–Planck equation, which describes the linear-noise approximation, where 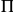 is a Gaussian. Note that the matrix entries 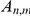 and 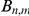 are not functions of 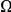. From Eq. (25) we obtain the time evolution of the first and second moments of the fluctuation,
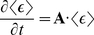
(28)

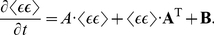
(29)Equations (28) and (29) are used in “Low confinement: the linear-noise approximation” in Section “Results” to analytically compute the autocorrelation function.

The solution of Eq. (28) with initial condition 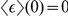 is 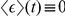. Hence
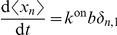


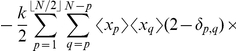


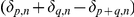


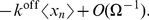
(30)I.e., in the linear noise approximation the mean obeys the macroscopic rate equation.
